# Unlocking genetic insights: Evaluating wheat RILs for physiobiochemical traits under terminal heat stress conditions

**DOI:** 10.1186/s12870-024-05062-z

**Published:** 2024-05-21

**Authors:** Mandeep Redhu, Vikram Singh, Anita Kumari, Renu Munjal, Shikha Yashveer, Somveer Nimbal, Ram Niwas, Swati Verma, Kritika Sharma, Atul Loyal, Rukoo Chawla, Rutuparna Pati, Chetan Singh, Mehdi Rahimi

**Affiliations:** 1 Department of Plant, Soil and Agricultural Systems, College of Agricultural, Life and Physical Sciences, Southern Illinois University, Carbondale, IL 62901 USA; 2 Department of Genetics and Plant Breeding, College of Agriculture, CCS Haryana Agricultural University, Hisar, 125004 India; 3 College of Botany & Plant Physiology, College of Basic Sciences and Humanities, CCS Haryana Agricultural University, Hisar, 125004 India; 4 Department of Molecular Biology, Biotechnology and Bioinformatics, College of Basic Sciences and Humanities, CCS Haryana Agricultural University, Hisar, 125004 India; 5 Department of Mathematics, College of Basic Science and Humanities, CCS Haryana Agricultural University, Hisar, 125004 India; 6 College of Agriculture, CCS Haryana Agricultural University, Hisar, 125004 India; 7 Department of Biotechnology, Institute of Science and High Technology and Environmental Sciences, Graduate University of Advanced Technology, Kerman, Iran

**Keywords:** Wheat, Biochemical, Physiological, Heritability, Skewness, Kurtosis, HSI

## Abstract

**Background:**

The increasing impacts of heat stress on wheat production due to climate change has entailed the development of heat-resilient crop varieties. To address this, two hundred recombinant inbred lines (RILs) derived from a cross between WH711/WH1021 were evaluated in a randomized block design (RBD) with two replications at CCSHAU, Hisar, during 2018-19 under heat stress and non-stress conditions. Heat stress was induced by altering the date of sowing so that the grain filling stage coincide with heat stress.

**Results:**

Heat stress adversely affects RILs performance, as illustrated by alterations in phenotypic traits. Highest coefficients of variations were recorded for TAA, CTD 1, WUE, CTD 2, Cc and A under non-stress and heat stress conditions whereas gs, WUEi and GY under non-stress and SPAD 1, SPAD 2, GY and NDVI 2 under heat-stress conditions recorded moderate estimates of coefficient of variations. CTD 2, TAA, E, WUE and A displayed a significant occurrence of both high heritability and substantial genetic advance under non-stress. Similarly, CTD 2, NDVI 2, A, WUEi, SPAD 2, gs, E, Ci, MDA and WUE exhibited high heritability with high genetic advance under heat-stress conditions.

**Conclusions:**

Complementary and duplicate types of interactions with number of controlling genes were observed for different parameters depending on the traits and environments. RILs 41, 42, 59, 74, 75, 180 and 194 were categorized as heat tolerant RILs. Selection preferably for NDVI 1, RWC, TAA, A, E and WUEi to accumulate heat tolerance favorable alleles in the selected RILs is suggested for development of heat resilient genotypes for sustainable crop improvement. The results showed that traits such as such as NDVI, RWC, TAA, A, E, and WUEi, can be effective for developing heat-resilient wheat genotypes and ensuring sustainable crop improvement.

## Background

Wheat is a predominant cereal crop that ensures paramount role in global food security by fulfilling dietary requirements of millions of people all over the world with an average yield of about 280 million tons, highest in the world and an average production of 2.9 tons per hectare in Asian continent excluding Russia [1]. The probable reasons for its large-scale cultivation and dependency lies behind its high agronomic adaptability, convenience in grain storage and diverse uses of its flour in the preparation of various food items [2]. Despite the substantial growth in wheat production over the decades, it is difficult to cope with the demands of growing population [3, 4]. Furthermore, climate change and elevated temperature above a threshold are detrimental to the normal growth and development, known as terminal heat stress, affecting the physiological and biochemical processes to a great extent [5, 6]. The global mean temperature has experienced a consistent rise in recent decades, and this trend can be attributed to uncontrolled and unorganized anthropogenic activities. For each 1^o^C increase in temperature, the global wheat yield reduction is increased by 6.0 ± 2.9% [7]. Climate change and elevated temperature above a threshold are detrimental to the normal growth and affects the physiological and biochemical processes of wheat to a great extent. Prasad et al. [8] reported that wheat is more sensitive to nighttime temperature increase than daytime comparatively. Night temperature between 20 and 23 ^o^C delayed the grain filling period by 3 to 7 days, resulting in a decreased overall crop yield. The results also showed that both short- and long-term stresses can significantly influence growth and yield processes when stress occurs at sensitive stages and decreased them. Likewise, another study reported a significant reduction in grain filling duration and grain weight with 32/22 ^o^C day/night temperatures as compared to 25/15 ^o^C [9]. Therefore, the development of heat resilient genotypes becomes crucial.

The complex interplay morphological and physio-biochemical factors in determining the wheat yield under terminal heat stress makes it a thrust area for research. While earlier research focused on morphological parameters such as wheat height, tillers, grain yield, biological yield, harvest index, grain volume, and seed density for developing heat tolerant wheat cultivars, however genetic gain has reached on plateau because these alone are insufficient for developing effective cultivars in present scenario without full exploitation of other traits like biochemical, multispectral indices and gaseous exchange parameters [10]. Therefore, involvement of physio-biochemical parameters with high heritability and genetic advance can be valuable for assessing susceptibility/tolerance of genotypes and effective selection as they are directly involved in heat stress mechanism [10]. Elevated temperature at grain filling stage disrupts wheat metabolic and physiological responses. The photosynthetic efficiency of heat sensitive Rubisco, a major photosynthetic enzyme, is compromised, impacting photoassimilates production [11]. Many traits, such as total antioxidant activity (TAA), canopy temperature depression (CTD), water-use efficiency (WUE), grain yield (GY), chlorophyll content (SPAD), normalized difference vegetative index (NDVI), stomatal conductance (gs), intercellular CO_2_ concentration (Ci), and malondialdehyde content (MDA) are affected by heat stress.

Stomal conductance is crucial determinate of carbon assimilation and transpiration in wheat (C_3_ plants) as it is directly related to regulation of Co_2_ movement in leaves [12]. High temperature disrupts plant-water relations, affecting physiological phenomena like, photosynthesis, chlorophyll content, water use efficiency, leading to yield losses [13]. Furthermore, the production of reactive oxygen species (ROS) under heat stress compromises cell membrane integrity, resulting in increase in electrolyte leakage and lipid peroxidation of polyunsaturated fatty acids, whose end product is malondialdehyde content (MDA) [14]. Under severe stress, plants start to change green to yellow color at phenotypic level, whereas, toxic ROS accumulate at cellular level, as a consequence, plants defense mechanism activates the production of antioxidant enzymes [15]. The tolerant genotypes are reported to express high antioxidant enzymatic activity on high production of MDA content, therefore, both traits can be important criteria for screening out heat susceptible/tolerant genotypes. Plants can experience some degree of relaxation when the canopy surface temperature is successfully lowered by a significant loss of water content [16]. Additionally, it is reinforced by a rise in transpiration rate, which lowers water use efficiency (WUE). Consequently, plant closes their stomata and impairs the stomatal conductance, therefore, involvement of these parameters during selection process can be very effective. Moreover, the heat susceptibility index (HSI) can be a useful tool for identifying heat tolerant genotypes under heat stress conditions [17]. In summary, the importance of creating genotypes of wheat that are heat-resistant in response to climate change and rising temperatures must be emphasized. Through the application of plant breeding and biotechnology, scientists can produce wheat varieties that are more suited to a warmer climate, which will guarantee future generations’ access to food, environmental sustainability, and economic viability.

During crucial growth stages like flowering and grain filling, wheat, a cool-season crop, is especially vulnerable to high temperatures. Prolonged heat stress causes physiological changes in wheat plants that can lead to reduced photosynthesis, impaired grain development, and decreased overall productivity. So, a two-tiered based approach including field level phenotyping of physio-biochemical and gaseous exchange traits and various degree statistics was employed to uncover the genetic insights of RILs population under terminal heat stress conditions and identifying the heat resilient genotypes which further can be directly utilized in heat tolerant wheat breeding programs.

## Materials and methods

The experiment material was composed of two hundred RILs derived from a cross between WH711 and WH1021 using single seed descent method. The female parent, WH711 is known for high yield under timely and irrigated conditions, whereas, male parent, WH 1021 is a heat tolerant cultivar suitable for late sown and irrigated conditions. The experimental material composed of a selected subset of 200 RILs of early and late duration was sown in a randomized block design (RBD) during *Rabi* season 2018–2019 at CCSHAU, Hisar. Hyperspectral physiological parameters included normalized difference vegetative index at anthesis (NDVI 1) and 15 days after anthesis (DAA) (NDVI 2), chlorophyll content (SPAD 1 and SPAD 2), canopy temperature depression (CTD 1 and CTD 2), and relative water content at anthesis stage. Biochemical parameters including lipid peroxidation malondialdehyde content (MDA) and total antioxidant activity (TAA) were estimated at anthesis stage using [18] and [19] methods. The gaseous exchange parameters including intercellular CO_2_ concentration (µmole/mole of air), transpiration rates (E) (mmol m^− 2^s^− 1^), stomatal conductance (gs) (mol m^− 2^s^− 1^), photosynthetic rates (A) (µmol m^− 2^s^− 1^), instantaneous water-use efficiency (WUE = A/E), carboxylation capacity (Cc = A/Ci) and intrinsic water-use efficiency (WUEi = A/gs) were calculated at anthesis stage using a portable infrared gas analyzer (IRGA). Descriptive statistics, including mean, genotypic and phenotypic coefficient of variations (GCV & PCV), heritability, genetic advance (GA), skewness, and kurtosis were computed using IBM SPSS statistics. The significance of skewness and kurtosis was determined by dividing these values by their respective standard errors, yielding t values that were compared against the t-table values at n-1 degrees of freedom for assessment. Heat susceptibility index (HSI) was calculated for all the traits within the RILs population utilizing the formula described by [20].

## Results and discussion

### Change in physio-biochemical and gaseous parameters

The differential response of RILs population provided a clear picture of impacts of terminal heat stress (Table 1). The differences in the heat tolerance of RILs population were most evident in the changes in grain yield per plot. Generally, high yielding cultivar is a main goal of all crop improvement programmes [21]. The hyperspectral physiological parameters recorded highest reduction for NDVI 2 (0.80 to 0.54) followed by NDVI 1 (0.82 to 0.72), SPAD 1 (49.41 to 41.55) and SPAD 2 (47.09 to 39.32) (Table 1). The intense loss of greenness and chlorophyll content (SPAD units) disrupted the photosynthetic machinery, A (15.70 to 7.26) and membrane permeability, RWC (79.92 to 77.26%) and forced the plants to complete its growing degree days while compensating the economic and biological yield. The analogous findings were reported by [16, 22]. The heat stress impacts were more dramatic in CTD and gaseous traits, such as a significant increase was recorded for CTD 1, CTD 2 and Ci whereas, significant reduction was observed for E, gs, Cc, WUE, and WUEi. Similarly, cooler canopy with reduced photosynthetic and transpiration rate were recorded in other findings [16, 23]. The impaired stomatal conductance and water loss directly hindered the photosynthates translocation and ultimately decreased the economic yield [16, 24]. Heat stress created enough stress to trigger the production of MDA content (from 0.15–1.13 to 0.15–2.38 µ mole/g fresh weight) and consequently, a differential response of TAA (from 5.11–30.75 to 10.10–45.20 µ mole/g fresh weight) was observed. Likewise other traits were also recorded with wide ranges, indicating towards the presence of ample amount of variations among RILs population, which can potentially provide some desirable genotypes. Earlier reports also observed severe reduction among different traits including antioxidant content, chlorophyll content, photosynthesis, stomatal conductance and transpiration under heat stress conditions [14, 15].

**Fig. 1:**
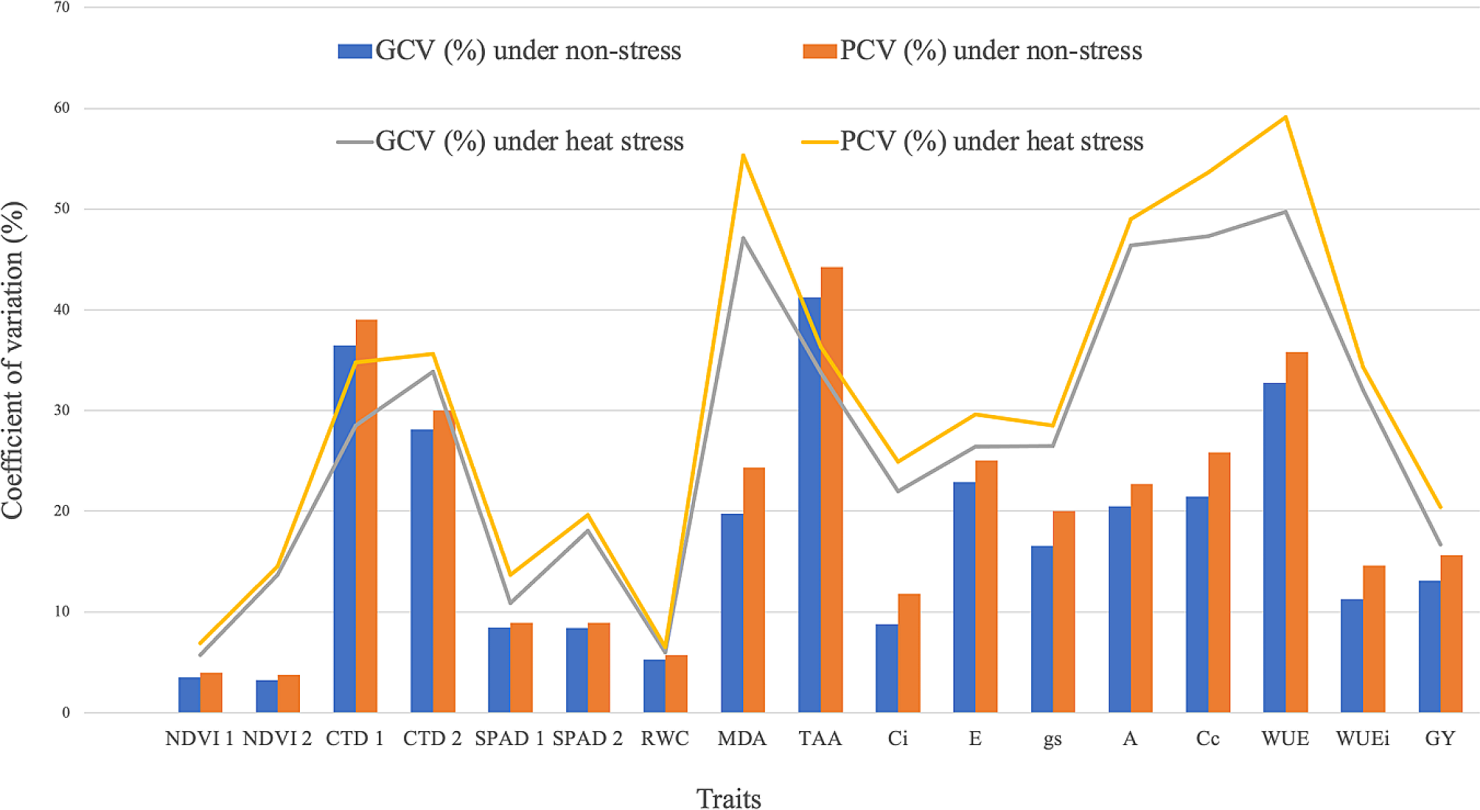
Estimate of GCV (genotypic coefficient of variations) and PCV (phenotypic coefficient of variations) for different physio-biochemical traits in RILs population under heat stress and non-stress conditions

### Coefficients of variability

The recorded traits showed slightly higher PCV compared to GCV, suggesting a predominance of genetic control in comparison to environmental factors (Fig. 1). Generally, higher values of GCV and PCV are preferred, as they provide a wider genetic base for the selection of desirable traits and result in an effective selection. High GCV and PCV values were observed for TAA (41.23% and 44.26%) followed by CTD 1 (36.48% and 39.03%) and WUE (32.78% and 35.78%) under non-stress conditions whereas, WUE (49.75% and 59.14%) followed by MDA (47.14% and 55.33%), Cc (47.32% and 53.62%), A (46.36% and 49.02%), CTD 2(33.83% and 35.62%) and TAA (33.78% and 36.36%) under heat stress conditions. However, NDVI 1(3.54% and 3.99%), NDVI 2 (3.28% and 3.75%), SPAD 1(8.45% and 8.92%), SPAD 2 (8.39% and 8.94), RWC (5.29% and 5.75%) and Ci (8.79% and 11.83%) under non-stress conditions and NDVI 1 (5.72% and 6.9%) and RWC (6.00% and 6.51%) under heat stress conditions recorded low values. Their low corresponding difference signified minimum difference between individual RILs, reflecting a higher level of acceptability for the results of these traits. Similar findings for canopy temperature and SPAD were reported by previous studies [25]. Rest of the traits showed moderate values for coefficients of variations, indicating that selection will be more responsive as compared to traits having low GCV and PCV values. These findings were aligned with other reports [26, 27].

### Estimates of heritability and genetic advance

Heritability estimates are a useful tool in gauging the authenticity of phenotypic selection by helping in identification of particular traits. In this study, high broad sense heritability was recorded for CTD 2 (88% and 90%) followed by SPAD 2 (88% and 85%), TAA (87% and 86%), RWC (85% and 85%), and A (81% and 89%) under both conditions, indicating that traits have potential to give a stable performance under heat stress as they are least affected by environmental factors (Table 1). These results aligned with the other findings [16, 28] reported high heritability estimates for photosynthetic rates, canopy temperature depression. Heat stress severely impacted heritability estimates for NDVI 1, CTD 1, SPAD 1, E and WUE, whereas, remaining traits like NDVI 2, MDA, Ci, gs, Cc and WUEi recorded the reverse trend. On other hand, TAA (79.11%), CTD 1 (70.22%), WUE (61.80%) and CTD 2 (54.15%) under non-stress conditions and A (90.34%), WUE (86.23%), Cc (86.04%), MDA (82.73%), TAA (64.65%) and gs (50.54%) under heat stress conditions recorded high estimate of GA as 5% of mean, indicating a good scope of improvement.

**Table 1:** Estimate of genetic parameters, and third degree statistics for different physio-biochemical traits in RILs population under heat stress and non-stress conditions

Traits	Range		Mean ± SE(m)		h^2^ (%)		GA (5% of mean)		Skewness		Kurtosis	
	Non-HS	HS	Non-HS	HS	Non-HS	HS	Non-HS	HS	Non-HS	HS	Non-HS	HS
**NDVI 1**	0.71–0.89	0.53–0.84	0.82 ± 0.03	0.72 ± 0.04	79	69	6.47	9.76	-0.18*	-0.37*	0.70*	0.07
**NDVI 2**	0.70–0.86	0.30–0.72	0.80 ± 0.03	0.54 ± 0.10	76	89	5.89	26.61	-0.83*	-0.11	0.80*	-0.43*
**CTD 1**	0.00-4.75	0.00–10.00	2.56 ± 0.60	6.08 ± 0.78	87	67	70.22	48.09	-0.30	-0.09	-0.71	-0.73
**CTD 2**	0.10–6.50	0.00–7.00	4.19 ± 0.60	4.78 ± 0.76	88	90	54.15	66.19	-0.27	-0.93	0.28	0.19
**SPAD 1**	40.00-60.90	21.2–61.5	49.41 ± 0.62	41.55 ± 0.67	90	63	16.48	17.74	0.47	0.35	-0.30	0.74
**SPAD 2**	34.50–55.80	21.1–57.5	47.09 ± 0.60	39.32 ± 1.22	88	85	16.23	34.23	-0.30	0.02	0.05	-0.53
**RWC**	59.87–85.18	50.82–82.96	79.92 ± 0.50	77.26 ± 0.45	85	85	10.04	11.41	-1.77*	-1.84*	3.05*	4.46*
**MDA**	0.15–1.13	0.15–2.38	0.63 ± 0.18	0.68 ± 0.45	66	73	33.13	82.73	0.17	1.25*	0.65*	0.84
**TAA**	5.11–30.75	10.10–45.20	14.21 ± 1.66	26.79 ± 1.81	87	86	79.11	64.65	0.89	0.11	0.20	-0.81
**Ci**	178.00-336.00	153–393	243.07 ± 1.49	285.62 ± 2.51	55	78	13.45	39.96	0.34	-0.24	-0.06	-0.30
**E**	3.00-7.99	0.23–6.99	5.20 ± 0.55	4.15 ± 0.61	84	79	43.23	48.45	0.38	0.48	-0.59	-0.36
**gs**	0.11–0.37	0.07–0.25	0.22 ± 0.09	0.13 ± 0.10	68	86	28.13	50.54	0.61*	0.61*	0.55*	0.19
**A**	9.57–25.08	3.21–21.69	15.70 ± 0.86	7.26 ± 1.32	81	89	38.08	90.34	0.67	1.93	-0.31	4.21*
**Cc**	0.03–0.13	0.01–0.14	0.07 ± 0.06	0.03 ± 0.10	69	78	36.74	86.04	0.82*	2.41*	0.36*	8.42*
**WUE**	1.44–7.89	0.65–6.49	3.22 ± 0.62	1.94 ± 1.06	84	71	61.80	86.23	1.37*	2.31*	2.34*	5.80*
**WUEi**	33.34–91.71	20.11–93.80	72.21 ± 1.08	55.61 ± 2.54	60	87	18.01	61.46	-0.14	0.25	-0.55	-1.14
**GY**	540–1220	217–900	855.05 ± 4.43	563.99 ± 4.50	70	67	22.58	28.18	0.13	0.20	-0.54	0.45

HS = Heat stress, NS = Non-stress, h^2^ = Heritability, GA = Genetic advance, NDVI 1 = Normalized difference vegetative index at anthesis, NDVI 2 = Normalized difference vegetative index at 15 days after anthesis, CTD 1 = Canopy temperature depression at anthesis, CTD 2 = Canopy temperature depression at 15 days after anthesis, SPAD 1 = Chlorophyll content at anthesis, SPAD 2 = Chlorophyll content at 15 days after anthesis, RWC = Relative water content, MDA = Lipid peroxidation malondialdehyde content, TAA = Total antioxidant activity, Ci = Intercellular CO_2_ concentration, E = Tanspiration rates, gs = Stomatal conductance, A = Photosynthetic rates, Cc = Carboxylation capacity, WUE = Instantaneous water-use efficiency, WUEi = Intrinsic water-use efficiency, GY = Grain yield per plot

Traits like NDVI 2, CTD 2, SPAD 2, MDA, Ci, gs, A, Cc, WUE, and WUEi recorded significant increase in GA under heat stress conditions, suggesting towards worthful results in case they are selected. The remaining traits showed decrease in GA values, suggesting that those traits were adversely affected by heat stress. Traits like NDVI 1, NDVI 2, SPAD 1 showed lower estimates of GA, indicated that these traits follow by non-additive / polygenic inheritance. Similar reports were recorded in previous findings [27, 29, 30].

### Genetics of traits

The skewness and kurtosis can be very instrumental to understand the distribution pattern of a traits by determining the gene action and nature of genes associated with the traits [31]. Complementary gene interaction is commonly associated with positive skewness, while duplicate gene interaction (additive X additive) is indicative of negative skewness. Additionally, platykurtic distributions result from the influence of a larger number of genes, whereas leptokurtic distributions are associated with the impact of a smaller number of genes.

**Fig. 2:**
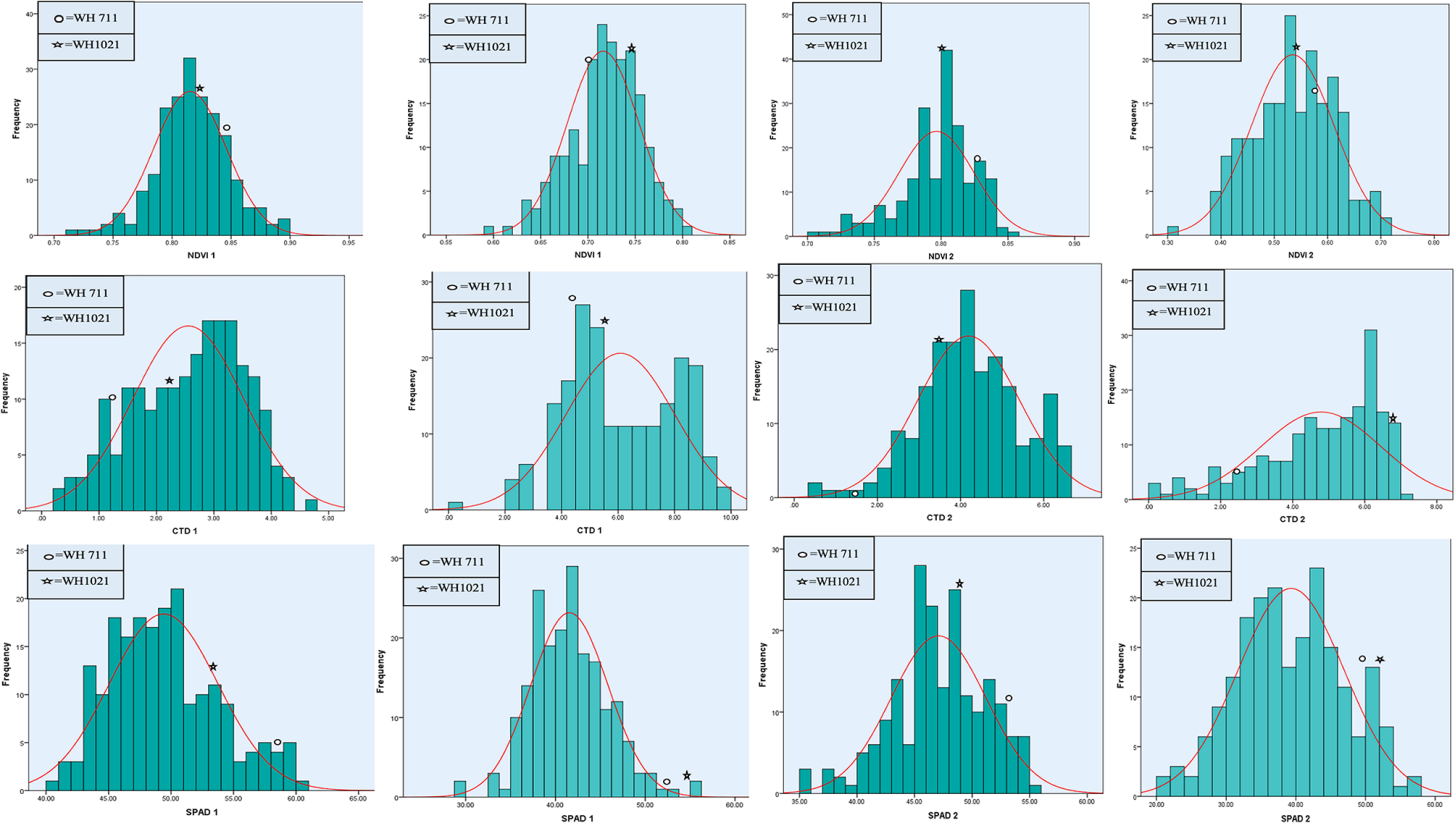
Frequency distribution of NDVI 1 (Normalized difference vegetative index at anthesis), NDVI 2 (Normalized difference vegetative index at 15 days after anthesis), CTD 1 (Canopy temperature depression at anthesis), CTD 2 (Canopy temperature depression at 15 days after anthesis), SPAD 1 (Chlorophyll content at anthesis) and SPAD 2 (Chlorophyll content at 15 days after anthesis) for RILs population and parents under non-stress (dark) and heat stress (light) conditions

**Fig. 3:**
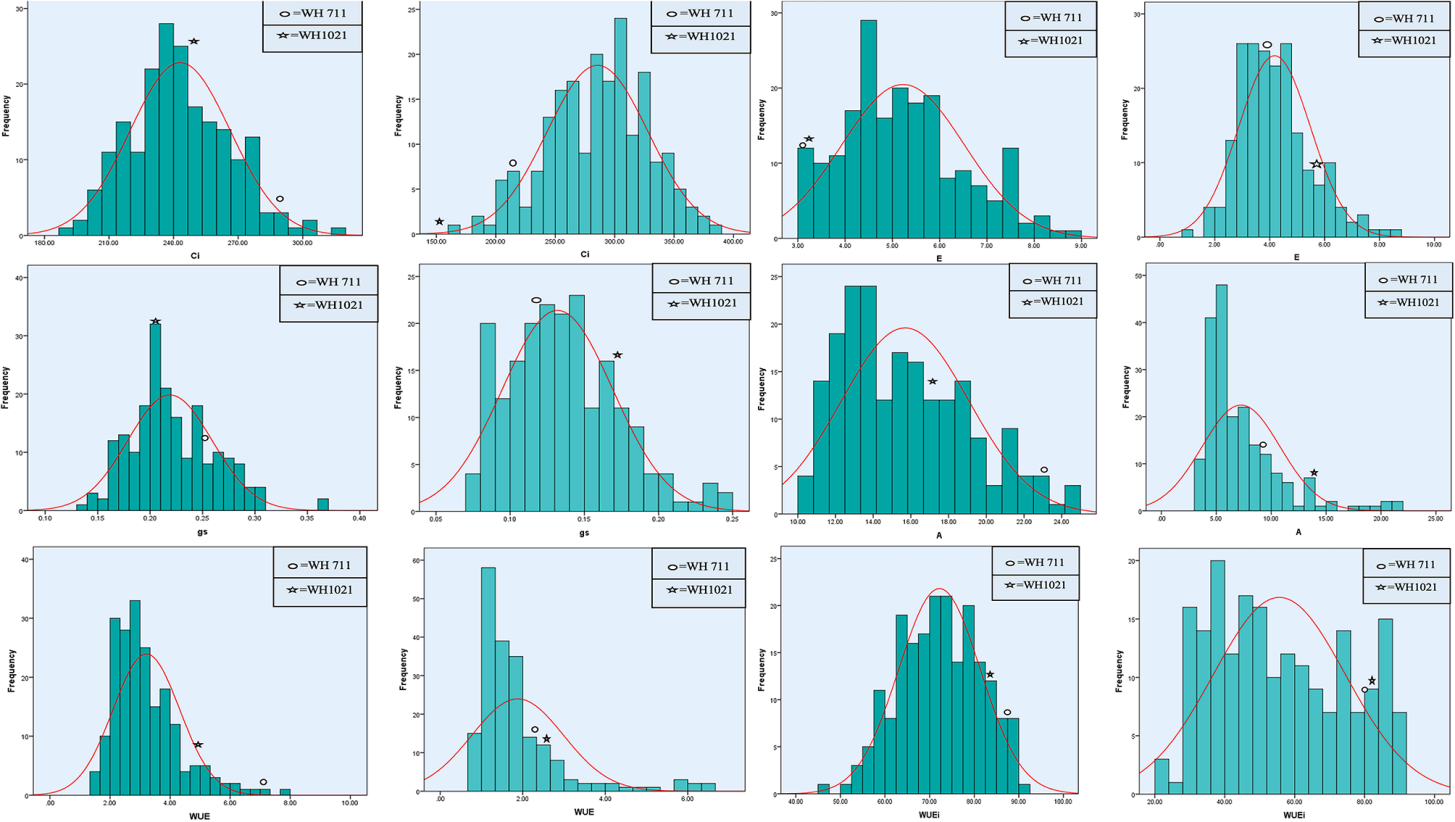
Frequency distribution of Ci (Intercellular CO_2_ concentration), E (Tanspiration rates), gs (Stomatal conductance), A (Photosynthetic rates), WUE (Instantaneous water-use efficiency) and WUEi (Intrinsic water-use efficiency) for RILs population and parents under non-stress (dark) and heat stress (light) conditions

**Fig. 4:**
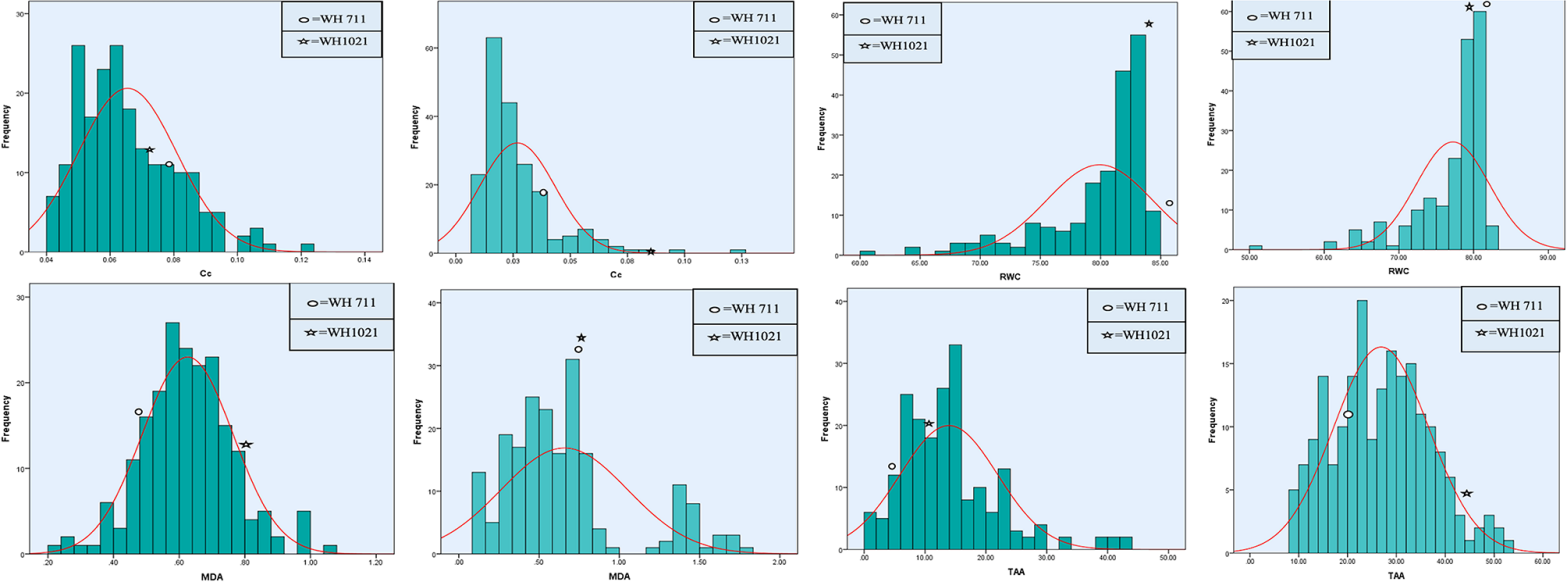
Frequency distribution of Cc (Carboxylation capacity), RWC (Relative water content), MDA (Lipid peroxidation malondialdehyde content) and TAA (Total antioxidant activity) for RILs population and parents under non-stress (dark) and heat stress (light) conditions

For most traits, the RILs exhibited a broad distribution that extended beyond the mean values of the parents for each trait, as illustrated in Figs. 2, 3 and 4. This suggests the presence of a significant amount of variability within the RILs population and their differential response for terminal heat tolerance. Among hyperspectral traits, NDVI 1 and NDVI 2 exhibited a significant negatively skewed (-0.18 and − 0.83) and positive kurtosis (0.70 and 0.80) under non-stress conditions, suggesting towards polygenic inheritance with increasing effects and dominance property in their expression. However, NDVI 1 exhibited only significant negative skewness and NDVI 2 exhibited only significant kurtosis under heat stress conditions. The genetic gain for NDVI 1 & 2 traits will be rapid under mild selection, performed based on existing variability in the RILs population. However, SPAD 1 recorded non-significant positive skewness with platykurtic distribution, indicating complementary and polygenic inheritance.

Among biochemical traits, MDA showed a significant positively skewness with platykurtic distribution, indicating presence of a large number of genes with increasing effects with dominance property. The genetic gain for MDA will be rapid under mild selection, performed based on existing variability in the RILs population. On the other hand, RWC exhibited negatively skewed (-1.77 and − 1.84) with a leptokurtic distribution (3.05 and 4.46) under both conditions, respectively, suggesting that, RWC followed a dominance based complementary (additive X additive) gene inheritance, controlled by lower number of genes with increasing effects. The stringent/ intense selection will be very effective and consequently, genetic gain will be highly responsive.

Among gaseous traits, heat stress severely impacted kurtosis distribution of gs by providing a nonsignificant (0.19) coefficients, whereas, skewness distribution (0.61 and 0.61) was constant under both conditions. The gs distribution prominently depicted dominant component, controlled by fewer number of genes, which will be responsive under mild selection. Likewise, skewness distribution of WUEi altered from negative to positive under nonstress to heat stress conditions. However, kurtosis distribution of A changed from platykurtic to leptokurtic while moving from non-stress to heat stress conditions, indicating that heat stress severely impacted functioning of some genes, governing A under non-stress condition and a few genes, controlling A were active under heat stress conditions. It is well proved fact that photosynthetic rate is the ultimate factor, affected by heat stress, as it burns the photosynthetic machinery and impaired the activity of heat sensitive Rubisco enzyme. Similarly, Cc and WUE showed a significant positively skewed (0.82 and 1.37 under non-stress, 2.41 and 2.31 under heat stress conditions) and platykurtic distribution 0.36 and 2.34 under non-stress conditions. However, kurtosis distribution was platykurtic under non-stress and leptokurtic under heat stress, indicating that some genes were inactive under heat stress conditions. The stringent selection will be effective for both traits.

### Selection of superior recombinant and traits contributing heat tolerance

The assessment of the top 10 and bottom 10 performing RILs underscored the existence of a transgressive segregant population across all traits. Different genetic interaction like dominant, additive and epistatic interactions might be responsible for the occurrence of transgressive segregants. The mean performance of top ten RILs showed significant achievement, even higher than the parents for most of the traits *viz.* NDVI 1 (0.88 and 0.79), NDVI 2 (0.84 and 0.69), CTD 1 (4.16 and 9.25), CTD 2 (6.36 and 6.85), SPAD 2 (54.61 and 53.76), MDA (0.95 and 1.63), TAA (29.75 and 44.86), Ci (294.90 and 364.65), E (7.73 and 6.85), gs (0.31 and 0.22), A (23.49 and 18.41), WUE (6.33 and 5.69) and WUEi (88.53 and 88.55) under non-stress and heat stress conditions, respectively (Table 2). In general, the generation of ROS during heat stress is a multifaceted but crucial component of plant defense mechanisms. In order to design crop varieties with increased resilience to environmental challenges and, eventually, contribute to sustainable agriculture and food security in a changing climate, it can be helpful to understand the role of ROS in signaling cascades and defense mechanisms. Based on highest GY under heat stress conditions, (895 g), RIL 59 can be categorized as heat tolerant RIL, which was further supported by highest TAA (59 µ mole/g fresh weight). The results show that antioxidant enzymatic activity and malondialdehyde content traits can be effective for screening heat-tolerant genotypes.

**Table 2:** Mean performance of ten highest and lowest RILs and parents under non-stress and heat stress conditions

Traits	Under NS				Under HS			
	Parental genotypes		RILs		Parental genotypes		RILs	
	WH711	WH1021	High	Low	WH711	WH1021	High	Low
**NDVI 1**	0.84	0.82	0.88	0.74	0.70	0.74	0.79	0.63
**NDVI 2**	0.82	0.80	0.84	0.72	0.59	0.55	0.69	0.39
**CTD 1**	1.26	2.30	4.16	0.61	4.50	5.47	9.25	2.19
**CTD 2**	1.50	3.50	6.36	1.44	2.50	6.75	6.85	0.62
**SPAD 1**	58.60	53.66	59.07	42.16	53.00	55.68	51.53	33.27
**SPAD 2**	53.10	48.95	54.61	37.63	50.00	52.00	53.76	24.23
**RWC**	87.24	83.87	84.15	67.03	75.10	80.43	82.13	63.71
**MDA**	0.68	0.55	0.95	0.33	1.02	0.74	1.63	0.22
**TAA**	12.86	23.18	29.75	5.76	24.66	43.48	44.86	10.55
**Ci**	288.00	247.50	294.90	201.05	215.00	200.00	364.65	196.85
**E**	5.20	4.90	7.73	3.12	3.89	5.84	6.85	2.09
**gs**	0.26	0.21	0.31	0.15	0.12	0.17	0.22	0.08
**A**	23.04	17.39	23.49	10.86	9.20	14.05	18.41	3.65
**Cc**	0.08	0.07	0.10	0.04	0.04	0.09	0.08	0.01
**WUE**	4.43	3.55	6.33	1.71	2.37	2.41	5.69	0.81
**WUEi**	88.62	84.88	88.53	53.68	80.15	82.65	88.55	26.73
**GY**	1050.00	905.00	1119.15	618.05	660.00	710.00	802.15	350.15

HS = Heat stress, NS = Non-stress, NDVI 1 = Normalized difference vegetative index at anthesis, NDVI 2 = Normalized difference vegetative index at 15 days after anthesis, CTD 1 = Canopy temperature depression at anthesis, CTD 2 = Canopy temperature depression at 15 days after anthesis, SPAD 1 = Chlorophyll content at anthesis, SPAD 2 = Chlorophyll content at 15 days after anthesis, RWC = Relative water content, MDA = Lipid peroxidation malondialdehyde content, TAA = Total antioxidant activity, Ci = Intercellular CO_2_ concentration, E = Tanspiration rates, gs = Stomatal conductance, A = Photosynthetic rates, Cc = Carboxylation capacity, WUE = Instantaneous water-use efficiency, WUEi = Intrinsic water-use efficiency, GY = Grain yield per plot

**Fig. 5:**
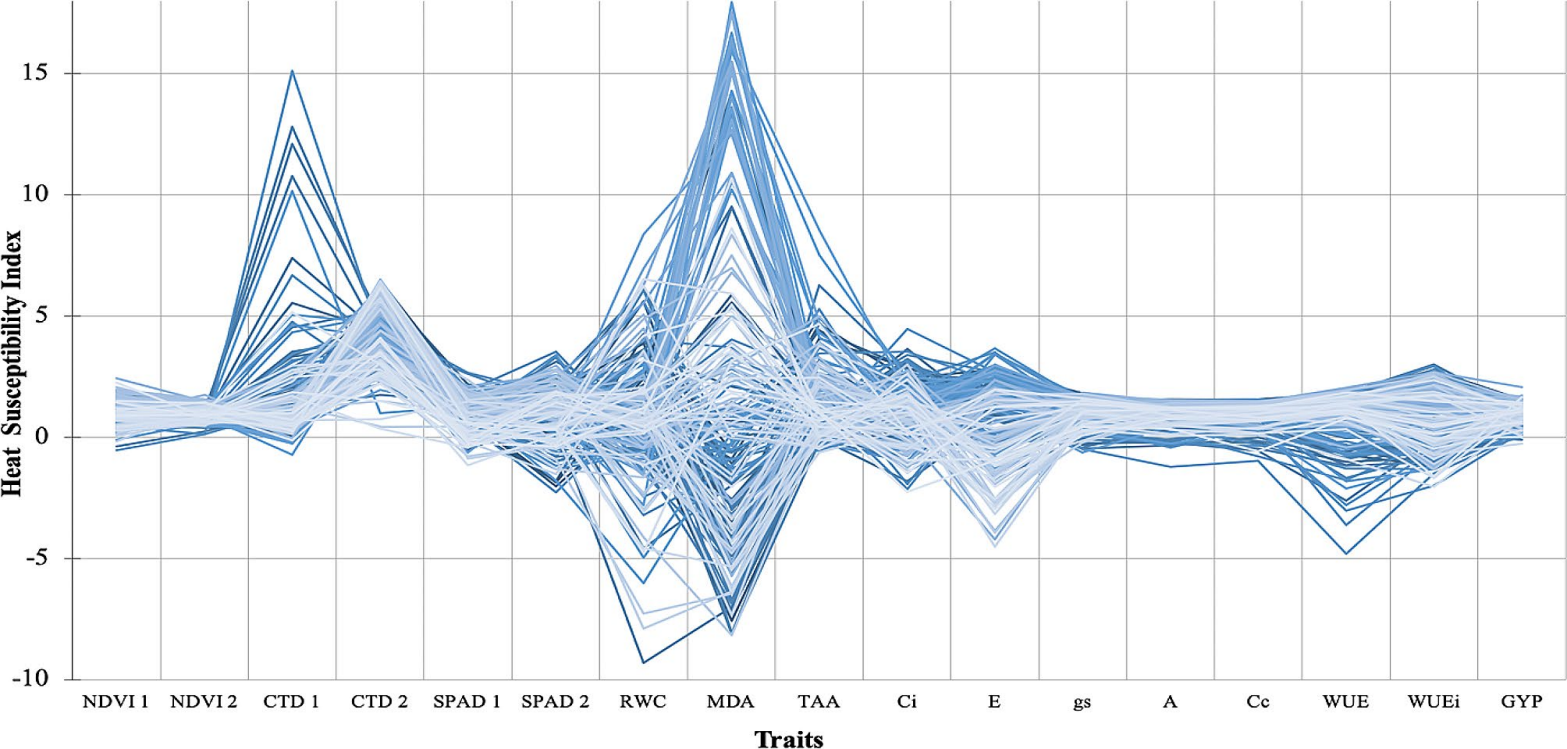
Distribution of Heat Susceptibility Index for different traits of whole RILs population. NDVI 1 = Normalized difference vegetative index at anthesis, NDVI 2 = Normalized difference vegetative index at 15 days after anthesis, CTD 1 = Canopy temperature depression at anthesis, CTD 2 = Canopy temperature depression at 15 days after anthesis, SPAD 1 = Chlorophyll content at anthesis, SPAD 2 = Chlorophyll content at 15 days after anthesis, RWC = Relative water content, MDA = Lipid peroxidation malondialdehyde content, TAA = Total antioxidant activity, Ci = Intercellular CO_2_ concentration, E = Tanspiration rates, gs = Stomatal conductance, A = Photosynthetic rates, Cc = Carboxylation capacity, WUE = Instantaneous water-use efficiency, WUEi = Intrinsic water-use efficiency, GY = Grain yield per plot

RIL 41 was recorded for highest A (21.32 µmol m-2s-1) and gs (0.25 mol m-2s-1), whereas, RIL 42 was recorded for highest CTD 1 (9.6 oC) and RWC (82.75%), however, both RILs had higher HSI values for GY (0.55 and 0.81), therefore, they can be considered as moderately tolerant. RIL 73 was recorded for maximum Cc (0.1264) with lowest Ci (163.5 µmol/ mole of air) and lower GY (526.50 g) under heat stress conditions. On other hand, RIL 77 exhibited moderate tolerance (0.72 HSI for GY), which was supported by maximum CTD 1 (9.6 ^o^C) with lowest E (1.65 mmol m^− 2^s^− 1^) under heat stress conditions. RIL 74 exhibited high tolerance (0.30 HSI for GY) with highest NDVI 2 (0.71), which was further supported by lowest HSI for NDVI 2 (0.12). Several investigations, including those conducted by [32, 33], identified heat-resistant genotypes through the screening of wheat populations under conditions of heat stress.

While calculating heat susceptibility indices (HSI) of individual trait, performed for whole RILs population, the lowest HSI values were recorded for RWC (-9.32) followed by MDA (-8.18) whereas, highest HSI values were recorded for MDA (17.94) and CTD 1 (15.12) (Fig. 5). Bennani et al. and Vijayalakshmi et al. [34, 35] observed the existence of transgressive segregants concerning heat stress indices. RIL 75 was recorded for lowest HSI values for maximum number of traits, including A (-1.23), Cc (-0.97), WUE (-4.81) and GY (0.13) which was further supported by maximum WUE (6.48); therefore, it can be termed as one of the highly heat tolerant genotype. RIL 194 also exhibited high heat tolerance (0.29 HSI for GY), which was supported by maximum SPAD 1 (56) under heat stress conditions with a lowest HSI (-1.16) for SPAD 1. RIL 180 was recorded for lowest HSI for GY, which was supported by lower HSI for different traits including NDVI 1 (0.33), SPAD 1 (0.10), SPAD 2 (0.43), RWC (-4.58), TAA (-0.15), E(0.38) and WUEi (0.72), whereas RIL 126 was recorded for highest HSI for GY, which was supported by higher HSI of different traits including NDVI 1(1.68), RWC (6.28), Ci (1.85), gs (0.99), A (1.43), cc (1.45), WUE (2.04) and WUEi (2.68), suggesting that the following physio-biochemical traits has a direct association and high potential for governing GY under heat stress conditions. In reaction to heat stress and water loss, plants may demonstrate several adaptation processes to survive with the tough conditions. These can include closing stomata to prevent water loss, creating protective chemicals such heat shock proteins, increasing antioxidant activity to buffer oxidative stress, and changing their metabolism to sustain important physiological activities. Overall, the relationship between water content loss, canopy surface temperature, and plant response to heat stress highlights the intricate balance that plants must maintain to survive under environmental pressures. In tough and ever-changing environmental conditions, plants require proper control of water and temperature.

An important tool for determining how sensitive a plant is to high temperatures is the Heat Susceptibility Index (HSI), which is a quantitative indicator of a plant’s susceptibility to heat stress based on physiological and biochemical responses. Researchers [36, 37] can use the HSI to compare the responses of different plant genotypes to high temperatures when examining their respective genotypes under heat stress; genotypes with lower HSI values are thought to be more heat-tolerant because they show less detrimental effects on growth, development, and physiological functions when exposed to heat stress.

The ability of the RILs to maintain their physiological and biochemical functions is a promising factor in alleviating the adverse effects of terminal heat stresses, as demonstrated by the current study. The RILs population exhibited a tendency to promptly respond to terminal heat stress through adjusting the level of greenness, relative water content, total antioxidant enzymes, transpiration rate, photosynthetic rate and intrinsic water use efficiency. This enables the plant to maintain a balance among physio-biochemical and gaseous exchange processes, leading to the high yield production. Hence, the present study suggests a comprehensive assessment of above-mentioned heat tolerant RILs (41, 42, 59, 74, 75, 180 and 194) through extensive trials under terminal heat stress target environments.

## Data Availability

Data are contained within the article.
